# Editorial: Biomarkers of health and disease in veterinary science, volume II

**DOI:** 10.3389/fvets.2026.1810540

**Published:** 2026-03-04

**Authors:** Alberto Muñoz-Prieto, Damián Escribano, Josipa Kuleš

**Affiliations:** 1 Interdisciplinary Laboratory of Clinical Analysis, Faculty of Veterinary Medicine, University of Murcia, Murcia, Spain; 2 Department of Chemistry and Biochemistry, Faculty of Veterinary Medicine, University of Zagreb, Zagreb, Croatia

**Keywords:** biomarker, clinical chemistry, omics, veterinary diagnostics, veterinary precision medicine

Biomarkers are extensively applied in veterinary medicine to evaluate health, support diagnosis and prognosis, guide and monitor therapy, and assess organ dysfunction (1). Across species, biomarkers bridge molecular, cellular, physiological, and ecological scales, offering insight into both individual health and population dynamics. The second volume of the Research Topic “*Biomarkers of health and disease in veterinary science*” brings together 13 original articles and perspectives that collectively reflect the expanding scope, methodological sophistication, and translational relevance of biomarker research in veterinary medicine. The contributions span multiple species, biological systems, and analytical approaches, illustrating how biomarkers are transforming both research and clinical practice.

Biomarkers offer more than diagnostic support. They enable the early identification of subclinical disease, provide insight into pathophysiological mechanisms, and facilitate the monitoring of disease progression and therapeutic response. Importantly, they also support a shift toward predictive, preventive, and personalized veterinary medicine, a concept increasingly aligned with advances in human biomedical research (2). However, veterinary science faces distinctive challenges: species diversity, environmental variability, differences in lifespan and metabolism, and limited reference data across many taxa. These complexities make the discovery and validation of robust biomarkers not only scientifically challenging but also critically important.

Several studies demonstrate that even conventional laboratory variables, when interpreted through a biomarker-oriented lens, can yield clinically meaningful insights. Clinical biomarkers remain central to patient monitoring, as shown by the study of intracaecal fluid therapy in horses (Ventura Lopes Carvalho et al.), which demonstrated that clinical signs, acid–base balance, and electrolyte profiles can safely guide alternative rehydration strategies when conventional routes are unavailable.

Similarly, rumen function indices and hepatorenal biomarkers in sheep with acute ruminal impaction (Elmeligy et al.) demonstrated that integrated biochemical panels can serve both diagnostic and prognostic roles, tracking recovery over time. In canine medicine, age-related variation in the Na:K ratio highlights the importance of age-specific reference intervals to avoid misinterpretation of endocrine or metabolic disease markers (Zemko et al.). Another comprehensive study challenges traditional views of equine metabolic syndrome by showing that hyperinsulinemia and metabolic dysfunction can occur independently of obesity, revealing a more complex metabolic landscape in horses (Aboelmaaty et al.). Its findings underscore the need to move beyond body condition alone and adopt biomarker-driven approaches for more accurate diagnosis and risk stratification.

At the population level, serum protein electrophoresis in free-ranging caribou (Daleo et al.) revealed strong seasonal influences on protein fractions, reinforcing the need for ecological context when using biomarkers in wildlife health monitoring.

Inflammation remains a core diagnostic target across veterinary species. The study of procalcitonin and C-reactive protein in dogs with sepsis and non-infectious systemic inflammatory response syndrome (SIRS) (Rompf et al.) demonstrated that while CRP is a reliable inflammatory marker, procalcitonin may serve better as a prognostic rather than diagnostic indicator in canine patients.

Expanding beyond companion animals, white blood cell estimates in endangered seabird Marbled Murrelets (Ryan et al.) demonstrated that simple hematologic biomarkers can reflect both individual physiological condition and broader ecosystem-level health trends, thereby supporting their inclusion in wildlife health assessment frameworks.

Stress-related immune modulation in camels has been investigated using serum cortisol as a biomarker (Hussen and Althagaf). Elevated cortisol levels were significantly associated with leukocytosis and altered neutrophil-to-lymphocyte ratios, providing a mechanistic link between stress and immune dysregulation. These findings further highlight the importance of considering gender and reproductive status when interpreting stress–immune interactions.

In ophthalmology, tear film parameters in French Bulldogs (Spornberger et al.) revealed subclinical evaporative dry eye features even in apparently healthy dogs, supporting the concept of breed-specific biomarker baselines.

At the same time, the Research Topic embraces the rapid expansion of omics technologies. The rapid evolution of analytical technologies has greatly expanded the range of putative biomarkers described in the literature and translated into clinical and research practice (3, 4). Whole blood fatty acid lipidomics in canine periodontal disease (Silva et al.) demonstrated systemic metabolic shifts accompanying oral inflammation, identifying specific fatty acids as candidate disease progression markers.

At the molecular level, targeted next-generation sequencing in German Shepherd dogs with inflammatory bowel disease (Peiravan et al.) refined previously identified GWAS loci and revealed single-nucleotide polymorphisms in immune-related genes. These findings advance genetic risk stratification in veterinary gastroenterology and highlight the potential of such markers for predicting therapeutic response in dogs with inflammatory bowel disease.

Cancer biomarker discovery is represented by metastasis-associated protein 1 (MTA1) in canine urothelial carcinoma (Campanelli et al.), where overexpression correlated with tumor aggressiveness and prognosis, positioning MTA1 as a promising molecular marker for veterinary oncology.

The critical need for improved “pain” biomarkers is highlighted in the perspective on acute and chronic pain in canine internal medicine (Sander et al.). The authors underscore the absence of objective, validated biomarkers for pain and advocate for multimodal diagnostic frameworks. The article reviews current approaches to pain assessment in dogs, including the reliance on subjective clinical pain scales, and discusses the inherent limitations and challenges of these tools in routine practice. It further outlines future directions for the development of novel, clinically applicable pain scales and individualized pain assessment strategies in canine internal medicine.

The expanding role of biomarkers in veterinary science will continue to strengthen translational links with human medicine and contribute to improved animal health and welfare. By bridging classical diagnostics with cutting-edge molecular approaches, they offer the promise of earlier detection, more targeted therapies, and improved outcomes for animals across diverse species and environments. Nevertheless, further multicenter validation studies, analytical harmonization, and standardization of reference values remain essential to ensure the clinical implementation of novel biomarkers across species and settings.

This Research Topic emphasizes that the future of veterinary diagnostics lies in multimodal biomarker platforms, species- and age-specific reference intervals, and translational approaches linking molecular data to clinical and population-level outcomes. As these tools continue to mature, they will play an increasingly vital role in advancing precision veterinary medicine and global One Health initiatives.
